# Safety in Rats of a Novel Nasal Spray Formulation for the Prevention of Airborne Viral Infections

**DOI:** 10.3390/pharmaceutics15020591

**Published:** 2023-02-09

**Authors:** Mirella Tanori, Michele Pitaro, Emiliano Fratini, Eleonora Colantoni, Angela Amoresano, Simona Celentano, Barbara Chiaramonte, Mariateresa Mancuso

**Affiliations:** 1 Laboratory of Biomedical Technologies, Italian National Agency for New Technologies, Energy and Sustainable Economic Development (ENEA), Via Anguillarese 301, 00123 Rome, Italy; 2 INBB–Biostructures and Biosystems National Institute, Viale delle Medaglie d’Oro 305, 00136 Rome, Italy; 3 Department of Chemical Sciences, University of Naples Federico II, Via Cinthia 26, 80126 Naples, Italy; 4 Istituto Nazionale per l’Assicurazione Contro Gli Infortuni sul Lavoro (INAIL), P.le Pastore 6, 00144 Rome, Italy

**Keywords:** respiratory viral infections, nasal spray, medical device, usnic acid

## Abstract

Hexedra+^®^ is a nasal spray containing hydroxypropyl methylcellulose, beta-cyclodextrin, and usnic acid. It has been developed with the aim of reducing the risk of transmission of airborne viral infections, with particular reference to influenza and COVID-19. As part of the preclinical development of the product, we carried out a study on thirty male Wistar rats divided into three study groups and treated with Hexedra+, an alternative formulation containing a double concentration of usnic acid (0.015% instead of 0.0075%) or saline solution. Products were administered at the dose of 30 μL into each nostril, three times a day for seven consecutive days by means of a micropipette. By the end of the treatment period, no significant changes were observed in body weight. Histological examination of nasal mucosa and soft organs did not show any significant difference in the three study groups. Serum transaminase level remained in the normal limit in all the animals treated. The serum level of usnic acid was measured in order to assess the absorption of the molecule through the nasal mucosa. By the end of the study period, the usnic acid serum level was negligible in all the animals treated. In conclusion, the safety profile of Hexedra+ appears favorable in the animal model studied.

## 1. Introduction

A recent European Parliament resolution states that respiratory infectious diseases still represent a considerable threat to society, with a huge burden in terms of human life and economy [1]. For example, the cost of influenza in US prior to the COVID-19 pandemic was estimated in 11.2 USD billion annually, with 3.7 million outpatient visits, 247,000 hospital admissions, 36,300 deaths and more than twenty million working days lost [2].

Between 2002 and 2003, a new disease called SARS (Severe Acute Respiratory Syndrome) affected 8098 people in 26 different countries, most of them in the Guangdong province of China. The epidemic caused 774 deaths in 17 different countries, corresponding to a fatality rate of 10% [3]. The etiologic agent was a new beta coronavirus called SARS-CoV, transmitted from bats to humans through intermediate hosts identified in some wild animals sold in Chinese markets, like civets and raccoons. The overall economic burden of SARS was estimated between 30 and 100 billion USD [4]. Someone said, prophetically, that this epidemic would have been the dry run for a larger calamity in the future.

Swine flu affected the American continent in 2009 [5]. The etiologic agent was a H1N1 strain of orthomyxovirus which infected 59 million people and caused 265,000 hospitalizations in US alone.

Between April 2012 and January 2020, MERS (Middle East Respiratory Syndrome), an interstitial pneumonia caused by another new coronavirus called MERS-CoV, affected 2519 people and caused 866 deaths, corresponding to a fatality rate of 34.3% [6]. These figures are not conclusive, since the epidemic is still ongoing. A study carried out in South Korea estimated that the economic burden of MERS in 2005 was $ 2.6 billion [7]. To the best of our knowledge, no data have been published on the economic burden of MERS in other countries.

On the 31st of December 2019, the Chinese Health Authorities announced a suspected increase of interstitial pneumonia in the Hubei province, and mainly in its capital Wuhan [8]. It was the beginning of the COVID-19 pandemic caused by a third new coronavirus called SARS-CoV-2. According to the World Health Organization (WHO), by the 29th of January 2023, 752,517,552 people have been affected by the disease with 6,804,491 deaths (fatality rate: 0.9%) [9]. So far, the economic burden of COVID-19 has been dramatic. In 2020, the gross domestic product (GDP) in the European Union dropped by 7.4% [10].

Experts believe that respiratory viral infections will be more frequent in the future due to the expansion of large urban centers, the increase of air traffic and the climate changes linked to global warming [11]. Large urban centers facilitate virus transmission due to the close interindividual contiguity [12]. Air traffic moves every day large masses from one continent to another [13]. Finally, climate changes may have a direct impact on virus viability and host responses [14]. In fact, low temperatures increase the stability of many viruses [15]. A dry climate decreases the host’s mucociliary clearance, whereas high relative humidity increases the permanence of viral particles in the air [16].

Interindividual transmission of pathogens depends on four basic mechanisms: direct contact between people; indirect contact through fomites (i.e., contaminated objects); droplets falling on mucous membranes; and aerosol inhalation [17]. Until recently, it was believed that respiratory viruses were transmitted by droplets [18]. These particles, characterized by a diameter > 100 μm, fall within 50 cm if the infected subject coughs or sneezes (20 cm if the infected subject speaks). Infection transmission depends on droplet deposition on the oral or nasal mucosa, as well as the ocular conjunctiva.

Extensive researches carried out on SARS-CoV-2 have highlighted the role of aerosol in interindividual transmission of the infection [19]. Significant viral loads have also been measured in the aerosol of patients with measles [20], influenza [21], SARS [22], and MERS [23]. Aerosol particles are characterized by a diameter < 100 μm (usually < 5 μm). They are produced not only by coughing and sneezing, but also by breathing, talking, singing, or playing wind instruments. Aerosol remains suspended in the air for more than 5 s (sometimes for hours) and can be inhaled through the respiratory tract (airborne transmission) [24]. Nasal sprays, which create a physical barrier on epithelial cells, can be a useful complement to personal protective equipment for the prevention of airborne viral infections. They could trap viral particles and eliminate them through the mucociliary clearance. A carrageenan-based nasal spray showed to be safe and effective in preventing the common cold [25]. A surfactant (lecithin phospholipid) was able to bind the SARS-CoV-2 receptor-binding domain (RBD) in an *in-silico* study, prompting the development of lecithin-based nasal sprays to decrease the risk of COVID-19 transmission [26]. A polysaccharide-based nasal spray prevented in vitro the infection of Vero cells by SARS-CoV-2 [27]. A nasal spray based on magnesium aluminum silicate significantly decreased the titers of SARS-CoV-2 Delta variant in a 3D model of human nasal mucosa without toxic effects [28]. Finally, a nasal spray was able to decrease by 62% the transmission rate of SARS-CoV-2 in a high-risk population of healthcare workers [29].

Hexedra+ is an innovative nasal spray based on hydroxypropyl methylcellulose (HPMC), beta cyclodextrin (b-CD), and usnic acid (UA). HPMC produces a hydrogel on the nasal mucosa which blocks the access to epithelial cells of viral particles [30], as well allergens [31]. B-CD has been added to the formulation in order to increase UA solubility [32,33]. However, this molecule has intrinsic antiviral properties that could potentially contribute to the overall efficacy of the product [34].

UA is a natural compound characterized by antibacterial and antiviral activities. It inhibits the growth of several Gram-positive bacteria, including Staphylococcus aureus [35] and Staphylococcus epidermidis [36]. Noteworthy, it is also effective against methicillin-resistant Staphylococcus aureus [37,38], several multidrug-resistant Gram-positive bacteria [39], and some Gran-negative bacteria, including vancomycin-resistant enterococci [37].

UA possess also a significant inhibitory activity against several strains of influenza virus [40,41]. Surprisingly, two recent in silico studies have shown a strong binding affinity of UA for residues of the SARS-CoV-2 Spike protein RBD (i.e., the region of the protein which binds to ACE2 on the epithelial cell surface) [42,43]. By binding SARS-CoV-2 Spike protein, UA could contribute to the retention of viral particles into the hydrogel produced by HPMC. UA and remdesivir (a product recently approved for the treatment of COVID-19) showed a similar activity in a SARS-CoV-2 binding assay [44]. Finally, an in vitro study performed on Vero E6 cells showed a significant activity of UA against three different strains of SARS-CoV-2, namely Wuhan, Delta, and Omicron [45].

UA inhibits oxidative phosphorylation in mitochondria without affecting the respiratory chain and ATP synthase [46]. These properties probably explain its broad-spectrum antibacterial activity and represent the rationale for the inclusion of UA in several slimming formulations sold in the past as over-the-counter products. However, high oral doses of UA for slimming purposes have been related to the development of hepatotoxicity in a limited number of subjects. For this reason, the US Food and Drug Administration (FDA) sent a warning letter, followed by the withdrawal of the products from the market in November 2001 [47]. On the other hand, the topical use of UA appears to be safe and well tolerated [48], making the product an appealing ingredient to be included in nasal spray formulations aimed at decreasing the risk of transmission of airborne infections.

It is well known that nasal formulations have been used for a long time to deliver active ingredients into the circulation through the nasal mucosa. Therefore, part of the UA included in the formulation of Hexedra+ could potentially reach the liver, raising concern about the possible risk of hepatotoxicity. For this reason, the main purpose of the present study was the evaluation of the safety profile of Hexedra+ administered intranasally for one week to Wistar male rats. In order to rule out the risk of hepatotoxicity, the plasma level of UA was assessed at the end of the treatment period by liquid chromatography-mass spectrometry (LC-MS).

## 2. Materials and Methods

### 2.1. Animals

Wistar male rats (body weight 200–300 g) were purchased from Charles River Laboratories (Lecco, Italy). Animals were housed under conventional conditions with ad libitum feeding and artificial 12 h light/dark cycle. Their general health status was monitored and, during the experiment, body weight was registered daily. The study was carried out according to the European Community Council Directive 2010/63/EU. It was approved by the local Ethical Committee for Animal Experiments of the ENEA, and authorized by the Italian Ministry of Health (883/2021-PR).

### 2.2. Study Procedures

Rats were randomized in 3 groups (10 for each group) treated with Hexedra+, an alternative formulation called H150 or physiological solution (PS). Hexedra+ is a nasal spray containing HPMC, b-CD, tocotrienols, and UA (0.0075%). H150 has the same composition of Hexedra+, the only difference being a double concentration of UA (0.015% instead of 0.0075%).

Products were administered at the dose of 30 μL into each nostril, three times a day for seven consecutive days by means of a micropipette. The dose of 30 μL was chosen according to the data provided in Table 1.

In summary, the average area of nasal mucosa is 160 cm^2^ and 14 cm^2^ in man and rat, respectively (i.e., 11 times wider in men compared to rat). Since the recommended dose of Hexedra+ is 2–3 sprays per nostril (i.e., 200–300 μL per nostril), the corresponding dose in rat should be 18–27 μL per nostril. Therefore, a dose of 30 μL is 10% higher compared to the upper limit of the above-mentioned range in rat.

### 2.3. Histology, Morphometric Analysis and Immunohistochemistry

The day after the last treatment, the organs designed for morphological analysis (liver, brain, heart, spleen, kidneys and lungs) were quickly removed and fixed in formalin 10%. The diagonal section of the liver, lung, and spleen as well as the longitudinal section of the kidney, brain, and heart was obtained and processed for light microscopy, i.e., embedded in paraffin, sectioned at 4 μm, and stained with hematoxylin and eosin (H&E).

Skulls were also removed, and after fixation in 10% formalin for 24 h, they were incubated in a decalcifying solution (5% formic acid/4% hydrochloric acid) for 4 days. Transverse sections of nasal cavity were trimmed as follows: level 1, immediately posterior to the upper incisor teeth; level 2, at the incisive papilla; level 3, at the second palatine ridge; and level 4, at the level of the first upper molar teeth [50]. The sections of nasal cavity were then processed for light microscopy.

Nasal cavity sections stained with H&E were analyzed for the presence of edematous changes. Both the thickness of the olfactory epithelium and the proportion of glandular layer (relative to the thickness of the entire wall of mucosa) were measured. These measurements were all focused on the olfactory epithelium from the nasal septum in the region of the dorsal meatus. The thickness was quantified as the average of ten measurements, five from the right side and five from the left side of the septum, in nasal cavity sections of PS (*n* = 7), Hexedra+ (*n* = 6) and H150 (*n* = 6) rats.

Sections (4 μm) of paraffin-embedded nasal mucosa were also prepared to evaluate eventual presence of an inflammation status. Briefly, sections were dewaxed with Heat Mediated Antigen Retrieval Solution (“HMARS”, Abcam, Germania, Germany), washed in water for 5 min, and peroxidase inhibited by incubation in 3% H_2_O_2_ for 10 min. After incubation with 5% bovine serum albumin (Santa Cruz Biotechnology, Santa Cruz, CA, USA) diluted in phosphate-buffered saline (PBS) for 30 min, they were incubated at 4 °C overnight with an antibody against ionized calcium-binding adaptor molecule 1 (Iba-1; Wako Pure Chemical Industries sections, Osaka, Japan, 1:500), a well-known marker of macrophage activation. After incubation with the secondary anti-rabbit antibody (Abcam, Cambridge, UK; 1:200), the antigen-antibody reaction was revealed by DAB detection kit (Dako, North America, Inc., Carpinteria, CA, USA). Finally, sections were counterstained with hematoxylin. Iba-1 quantification was performed counting manually by the NIS-Elements BR 4.00.05 software (Nikon Instruments S.p.A., Florence, Italy) and the positive cell density was expressed as activated macrophages per area (mm^2^).

### 2.4. Enzyme-Linked Immunosorbent Assay (ELISA)

From each rat, a blood sample was collected into serum separator tube. After clot formation, serum was obtained by centrifugation (3000× *g* 15 min at 4 °C) and stored at −20 °C for the subsequent assay of alanine aminotransferase (ALT). Quantitative measurement of ALT protein was performed in duplicate by ELISA assay (Rat ALT SimpleStep ELISA^®^ Kit; Abcam) according to the manufacturer’s instructions.

### 2.5. Liquid Chromatography-Mass Spectrometry

Serum UA concentration was measured by precipitating 200 μL of serum with 600 μL of methanol. Samples were centrifugated for 10 min at 10000 rpm. Then, the supernatant was dried and resuspended in 100 μL of methanol. Using an Agilent 6420 Triple Quadrupole LC-MS/MS system with a HPLC 1100 series binary pump (Agilent Technologies, Waldbronn, Germany), 1 µL was analyzed by LC-MS/MS. The analytical column was a Phenomenex Kinetex 5 µm 100 A C18. The mobile phase was generated by mixing eluent A (0.1% Formic Acid in water) and eluent B (0.1% Formic Acid in methanol) for negative polarity. The flow rate was 0.2 mL/min. Elution gradient was from 20% to 80% solvent B in 5 min. Standard mixtures of 100 mg/L was diluted in MeOH/H_2_O 50:50. The calibration standards of 0.25–0.5–1–5–25–50–100 µg/L were prepared by serial dilution. Tandem mass spectrometry was performed using a turbo ion spray source operated in mode, and the multiple reaction monitoring (MRM) mode was used for the selected analytes. Extracted mass chromatogram peaks of metabolites were integrated using MassHunter Quantitative Analysis software rev. B.05.00 (Agilent Technologies, Santa Clara, CA, USA).

### 2.6. Statistical Analysis

Statistical tests were performed with GraphPad Prism software v.7 (GraphPad, San Diego, CA, USA). *p* values were determined using a two-tailed *t* test; * *p* < 0.05; ** *p* < 0.01; *** *p* < 0.001; **** *p* < 0.0001. Data were expressed as mean ± standard deviation (SD).

## 3. Results

### 3.1. Body Weight

**Figure 1:**
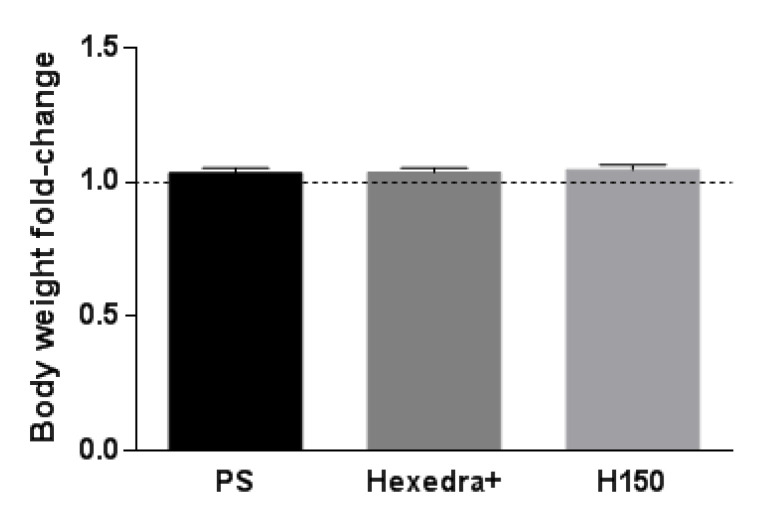
Rat body weight. Body weight change at the end of treatment with respect to baseline in rats treated with Hexedra+ compared to the alternative formulation with double quantity of UA (H150) and physiological solution (PS).

The general health status of animals was monitored during the experiment and the body weight was measured every day. Figure 1 shows the mean values of body weight at day 8 of treatment compared to baseline for each rat. No significant difference was observed in rats treated with Hexedra+ compared to H150 and PS.

### 3.2. Histological Analysis

**Figure 2:**
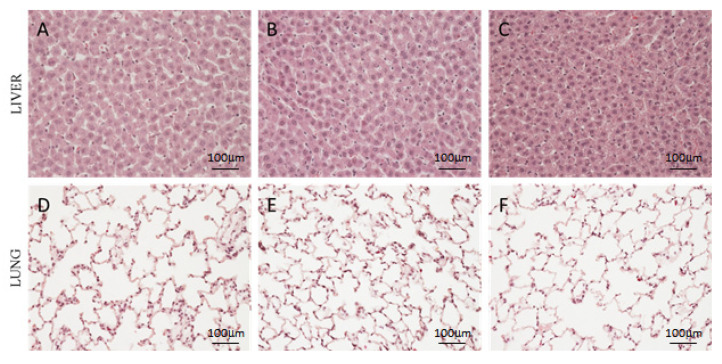
Histological analysis of rat liver and lung. Representative images of liver (**A**–**C**) and lung (**D**–**F**) of rats treated with PS (**A**,**D**), Hexedra+ (**B**,**E**) and H150 (**C**,**F**). PS: physiological solution; H150: alternative formulation with double quantity of UA.

At the macroscopic level, no variation in terms of size and color were observed in all soft organs (liver, brain, heart, spleen, kidneys and lungs) collected from rats treated with Hexedra+ compared to H150 and PS. At the microscopic level, all organs showed a normal architecture, with an absence of inflammatory infiltrate as well as of any type of positive and/or negative adaptation (hypertrophy, hyperplasia, metaplasia and dysplasia) typically associated to tissue injury. Furthermore, no signs of reversible (swelling or enlarged size) or irreversible (apoptosis and necrosis) cell injury were detected. Representative images of liver and lung sections are shown in Figure 2.

In the nasal mucosa, three types of epithelia were analyzed in depth, in order to exclude any possible tissue reaction after in situ Hexedra+ application: squamous, respiratory, and olfactory epithelia.

**Figure 3:**
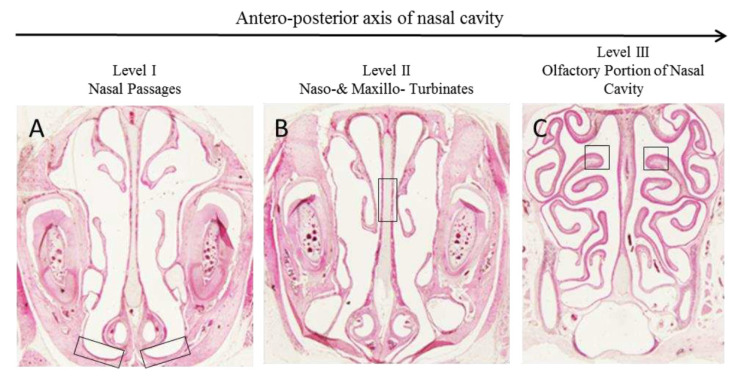
Distribution of the different types of epithelium within the nasal cavity of rats. At level I (**A**) of nasal passages is present the squamous epithelium; at level II (**B**) in naso- & maxillo turbinates there is the respiratory epithelium; finally, level III (**C**) is lined with the olfactory portion of nasal cavity. Boxes highlight the tract of epithelia analyzed.

The squamous epithelium (boxed in Figure 3) has an essentially defensive function, protecting the underlying tissues from the potential action of exogenous agents.

**Figure 4:**
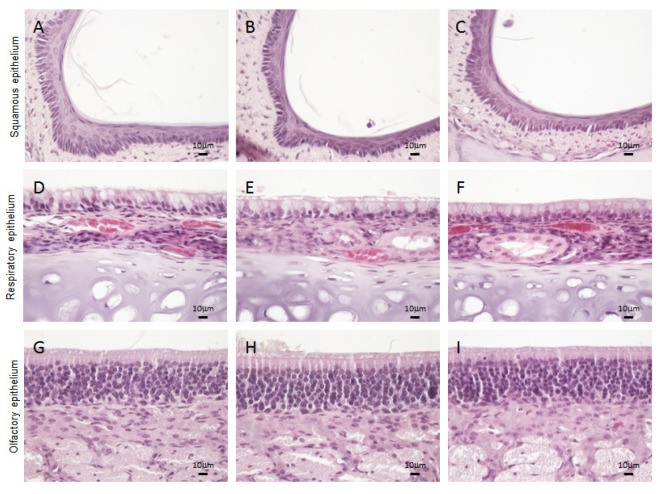
Histological analysis of nasal mucosa. Representative images of squamous (**A**–**C**), respiratory (**D**–**F**) and olfactory epithelium (**G**–**I**) of rats treated with PS (**A**,**D**,**G**), Hexedra+ (**B**,**E**,**H**), H150 (**C**,**F**,**I**). PS: physiological solution; H150: alternative formulation with double quantity of UA.

Similarly to rats treated with PS and H150, the squamous epithelium of rats treated with Hexedra+ appeared lightly keratinized and stratified, consisting of a layer of basal cells and a few layers of squamous cells that become flatter toward the surface (Figure 4A–C).

The respiratory epithelium includes four main cell types: ciliated, nonciliated, basal, and goblet cells. Since the density of these cells changes depending on the location of the respiratory epithelium in the nasal cavity, to avoid mistakes in the evaluation of their distribution level, the same tract of epithelium was analyzed for each sample (boxed in Figure 3B). The mucociliary compartment of the animals treated with Hexedra+ was well-organized, with the ratio of goblet cells to ciliated cells similar among groups. No inflammatory phenomena were observed in the submucosa (Figure 4D–F).

Finally, the olfactory epithelium of the nasal cavity was analyzed (boxed in Figure 3C). Three types of cells compose the olfactory epithelium: olfactory sensory neurons, supporting cells and basal cells. In rats treated with Hexedra+, the nuclei of all cells were aligned from the basal lamina to the apical surface similarly to the counterpart treated with PS or H150. In addition, Bowman’s glands and their respective ducts, extending from the lamina propria to the epithelial surface, did not show alterations (Figure 4G–I). Moreover, no inflammatory cells were observed in the submucosa.

### 3.3. Morphometric Changes and Inflammation Assessment in the Olfactory Mucosa

**Figure 5:**
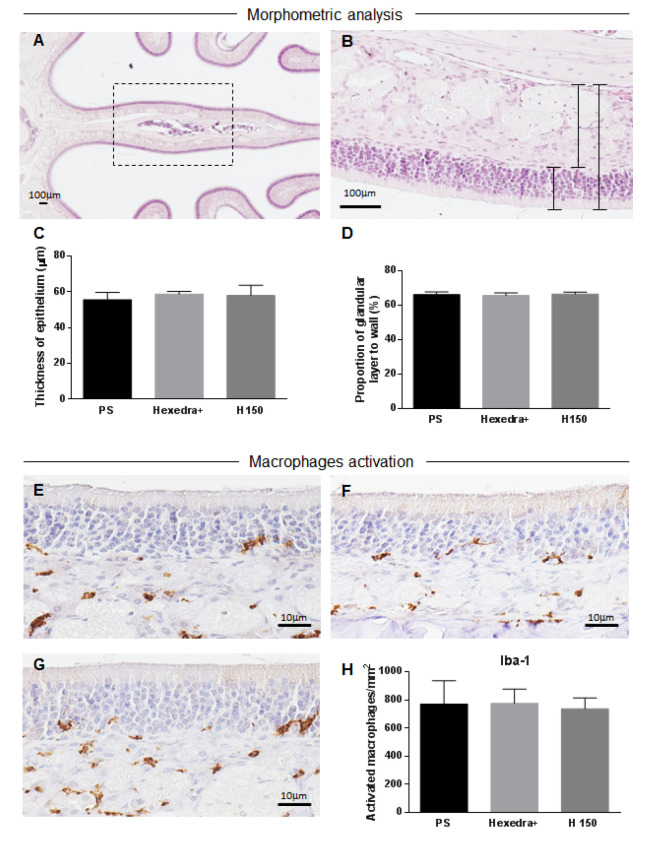
Morphometric analysis and macrophages activation in the olfactory mucosa. Representative image of nasal septum in the region of the dorsal meatus (dashed box) considered for measurements (**A**) and, at higher magnification, image of the thickness of olfactory epithelium and glandular layer (black lines in (**B**). Graphs with comparisons between treated and control groups for thickness of epithelium (**C**) and proportion of glandular layer to entire mucosa (**D**). Representative images of activated macrophage marker Iba-1 in the olfactory mucosa of rats treated with PS (**E**), Hexedra+ (**F**) and H150 (**G**) and relative quantitative representation (**H**). PS: physiological solution; H150: alternative formulation with double quantity of UA.

To further investigate the presence of inflammation and edematous changes in the olfactory mucosa of the nasal cavity, both the thickness of epithelium and glandular layer and the expression of the activated macrophage marker Iba-1 were analyzed. To evaluate morphometric changes of the olfactory mucosa, the nasal septum in the region of dorsal meatus of the nasal passage was considered, as highlighted in Figure 5A.

Both treated groups showed no significant differences in the thickness of epithelium (PS: 55.54 µm ± 4.14; Hexedra+: 58.71 µm ± 1.51; H150: 57.98 µm ± 5.73) (Figure 5B,C) and in the glandular proportion of the mucosa (PS: 66.29% ± 1.6; Hexedra+: 65.83% ± 1.47; H150: 66.67% ± 1.03) (Figure 5B,D) compared to the control group. Furthermore, immunohistochemical analysis of Iba-1 revealed no difference in the activation of macrophages in the olfactory mucosa of treated groups with respect to control group (Figure 5E–H).

### 3.4. Serum ALT

**Figure 6:**
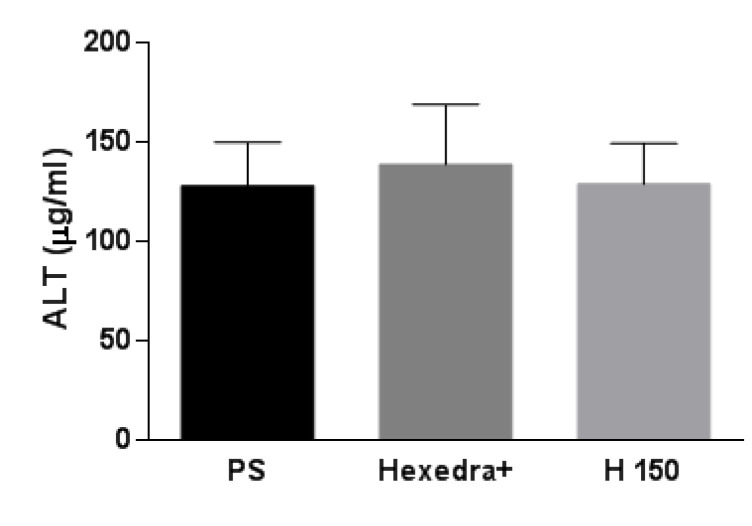
Rat serum ALT. Evaluation of ALT concentration level in rats treated with Hexedra+ compared to H150 and PS. PS: physiological solution; H150: alternative formulation with double quantity of UA.

ALT level is considered a sensitive and specific preclinical and clinical biomarker of hepatotoxicity [51]. However, an increase in serum ALT has also been associated with other organ toxicities, thus indicating that the enzyme has specificity beyond the liver in the absence of correlative histomorphological alteration in the liver. Results obtained from the ELISA assay (Figure 6) show that ALT activity was not statistically different in the three study groups.

### 3.5. Serum UA

Table 2 shows the serum concentration of UA in rats by the end of the study treatment. As expected, UA concentration was higher in rats treated with H150 (0.116 μM ± 0.006) compared to Hexedra+ (0.035 μM ± 0.004), while no UA was found in serum of rats treated with PS.

## 4. Discussion

Hexedra+ is an innovative nasal spray based on HPMC, b-CD, and UA aimed at blocking the access of viral particles, as well as allergens, to epithelial cells of nasal mucosa HPMC and b-CD are known to be safe and well tolerated. The former is currently used as an excipient in spray formulations for the administration of active ingredients through the nasal route [52]. The latter is also widely used as an excipient in several pharmaceutical formulations [53].

Lichen extracts with a high content of UA have been used for a long time in traditional medicine [54]. The first documented use of UA dates back to the first century BC. when dried lichen extracts were officially included in traditional Chinese medicine texts. The product was used for the treatment of malaria, wounds, and snake bites at the recommended dose of 6–9 g of dried lichen taken as a tea or decoction, corresponding to a daily dose of 60–120 mg of UA. Currently, UA is widely used in cosmetics, deodorants, toothpaste and medicinal creams, and studies published so far on the topical use of UA (mainly for the treatment of skin ulcers) have demonstrated a good safety and tolerability profile.

On the other hand, UA deserves a particular attention, due to the development in the past of hepatotoxicity in a limited number of subjects treated orally for slimming purposes. The content of UA in these formulations was particularly high, ranging from 600 to 1300 mg daily (around ten times the daily dose described in traditional medicine) [46]. Biopsy performed in the subjects who developed liver failure showed acute hepatocellular necrosis and inflammation, with a marked elevation of alanine aminotransferase (ALT) and minimal increase in alkaline phosphatase levels.

Due to the small dimension of the molecule, UA administered intranasally could potentially enter the blood circulation and reach the liver. For this reason, we performed an in vivo study in order to identify any possible damage to the nasal mucosa and other systemic districts (mainly liver, brain, and lungs). Our study demonstrated that Hexedra+ administered in rats by the nasal route three times a day for seven consecutive days is safe and well tolerated, without significant changes in body weight and damage or inflammation in nasal mucosa. The morphological analysis of all organs did not highlight visible alterations. In particular, no signs of hepatotoxicity were observed in any of the animals treated. Serum ALT levels at the end of the treatment period were in the normal range, with no statistical differences in the three study groups.

UA serum concentrations at the end of the treatment period were negligible and well below the concentrations found to be toxic for hepatocytes in toxicology studies. H150 (the alternative formulation containing twice the amount of UA) produced a UA serum level below the toxic concentrations as well. Noteworthy, in vitro toxicology studies have shown that UA concentrations < 1 μM are safe and well tolerated, while concentrations > 5 μM are toxic for hepatocytes [46]. To the best of our knowledge, this is the first assessment of UA absorption through the nasal mucosa into the circulation.

## 5. Conclusions

Hexedra+ is safe and well tolerated in rats after one week of treatment by the nasal route of administration. The treatment did not induce any change in body weight, nor damage or inflammation to the nasal mucosa. UA serum concentrations following the absorption of the molecule through the nasal mucosa were negligible and well below the concentrations found to be toxic for hepatocytes. Product safety was confirmed by the maintenance of a normal ALT level and the absence of any sign of liver damage, not only in rats treated with Hexedra+, but also in animals treated with an alternative formulation containing double the concentration of UA.

## Figures and Tables

**Table 1:** Nasal cavity characteristics in different species [49]. The volume to be administered has been adjusted according to the maximum recommended dose of Hexedra+ (300 μL per nostril).

Species	Surface of Nasal Mucosa	Volume of Nasal Cavity	Volume to Be Administered
Man	160 cm^2^	20 mL	300 μL
Rat	14 cm^2^	0.4 mL	26 μL
Mouse	2.8 cm^2^	0.03 mL	6 μL

**Table 2:** Serum UA concentration. Analysis of UA levels in serum from each rat by the end of the study treatment (data are expressed in μM). PS: physiological solution; Hexedra+; H150: alternative formulation with double quantity of usnic acid.

PS				Hexedra+				H150			
No.	S1	S2	Av.	No.	S1	S2	Av.	No.	S1	S2	Av.
1	<1	<1	<1	1	0.032	0.034	0.033	1	0.115	0.111	0.113
2	<1	<1	<1	2	0.044	0.036	0.040	2	0.121	0.116	0.119
3	<1	<1	<1	3	0.039	0.040	0.039	3	0.112	0.107	0.109
4	<1	<1	<1	4	0.031	0.036	0.034	4	0.126	0.117	0.121
5	<1	<1	<1	5	0.030	0.028	0.029	5	0.116	0.119	0.117
6	<1	<1	<1	6	0.030	0.040	0.035	6	0.126	0.119	0.123
7	<1	<1	<1	7	0.033	0.036	0.034	7	0.114	0.107	0.111
8	<1	<1	<1	8	0.033	0.034	0.034	8	0.135	0.109	0.122
9	<1	<1	<1	9	0.033	0.028	0.031	9	0.112	0.102	0.107
10	<1	<1	<1	10	0.032	0.042	0.037	10	0.120	0.111	0.116
Av.			<1	Av.			0.035	Av.			0.116
SD			n.a.	SD			0.004	SD			0.006

## Data Availability

Not applicable.
